# An Analysis of Small-Ruminant Farming in Marginal Area of the Mediterranean Region: A Focus on the Gentile di Puglia Breed

**DOI:** 10.3390/ani16091356

**Published:** 2026-04-28

**Authors:** Rosaria Marino, Mariangela Caroprese, Marzia Albenzio

**Affiliations:** Department of Agriculture, Food, Natural Resources and Engineering (DAFNE), University of Foggia, 71121 Foggia, Italy; mariangela.caroprese@unifg.it (M.C.); marzia.albenzio@unifg.it (M.A.)

**Keywords:** Mediterranean countries, small ruminants, biodiversity conservation, ecosystem services, Gentile di Puglia breed

## Abstract

This review provides insights on livestock farming in the Mediterranean regions, highlighting strengths, weakness, opportunities and threats. Biodiversity conservation and production of high-value, certified products are the main strengths of Mediterranean livestock systems. On the contrary, depopulation, low productivity and poor infrastructure and dependence on public subsides represent the main weaknesses of these systems. Climate change, market volatility and competition with intensive animal rearing systems are threats for Mediterranean livestock farming. A significant opportunity for Mediterranean livestock farming is represented by the presence of drought-tolerant native breeds and ecosystem services that contribute both to agricultural productivity and to ecosystem resilience and socio-cultural activities. Strategies that can promote local animal production in the Mediterranean region are provided with a focus on the Gentile di Puglia breed.

## Simple Summary

The Mediterranean area is a hotspot for traditional livestock farming systems. Sheep and goat husbandry have long been central to this area’s agrarian identity, producing high-quality products. The identification of the interactions between the key features of the livestock farming systems in the Mediterranean area and the possible strategies for its development represent a challenge and an opportunity for these rearing systems. Local breeds are precious genetic resources, adapted to the local environment and able to provide meat, milk, wool, and cultural ecosystem services. This review surveys livestock farming in the Mediterranean region, analysing recent trends and challenges related to local animal production and local breeds of the Mediterranean region with a focus on the Gentile di Puglia breed.

## 1. Introduction

The Mediterranean region is one of the oldest and most flourishing areas of agriculture and husbandry. Over thousands of centuries, the oro-geological movements have created a series of chains of mountains. autonomous marine basins, and a multitude of islands and peninsulas. As a consequence, a sort of “Mediterranean entity”, constituted by a mosaic of plant and animal populations stratified both in the maritime and mountainous zones affecting, directly, agriculture and livestock husbandry, resulted [1,2,3]. Indeed, the Mediterranean area shows great landscape heterogeneity due to human activities such as cultivation, wood harvesting and grazing [4,5]. These practices have been integral to preserving the cultural landscape and its associated ecological benefits, for example preventing the encroachment of woody vegetation and creating valuable habitats for local biodiversity [6]. Sheep and goat husbandry have always had a key role in agrarian identities of this area, providing food products, and contributing to territorial cohesion and biodiversity conservation, particularly in marginal and mountainous areas where crop farming is not viable [7]. In the last year, the Mediterranean scenario has changed, as reported by several authors, showing a decline of extensive livestock grazing, particularly throughout the Mediterranean mountain region [7,8,9,10]. Recently, sheep and goat biodiversity was studied and mapped in the Apulia region to preserve animal biodiversity and develop the regional economy [11]. Gentile di Puglia, literally “Gentle Apulian” due to its fine wool, is a millenary local sheep breed of the Southern Italy territory. For centuries, this breed was a symbol of transhumant livestock management [12]; after the Second World War, it was the dominant sheep breed in Southern Italy, with a population reaching almost 1 million animals [13]. Subsequently, many factors contributed to a dramatic reduction of the Gentile di Puglia herds. In recent decades, a recovery project was initiated with two purposes: (i) re-establish and conserve the original purebred populations, and (ii) improve the quality of milk, meat and wool productions [14,15].

The main purpose of the present review was to provide insights on livestock farming in marginal areas of the Mediterranean regions, highlighting strengths, weakness, opportunities and threats. A further goal is to propose strategies that can promote local production in the Mediterranean region by exploring a case study of the Gentile di Puglia breed.

## 2. Review Methodology

To conduct this review, a literature analysis was performed using the Google Scholar^®^, PubMed^®^, and Scopus^®^ databases, focusing on relevant peer-reviewed articles published in English only (mainly between 2014 and February 2026) to capture the most recent trends and challenges in livestock farming in the Mediterranean area. The key search terms used in various combinations were: “livestock farming systems AND Mediterranean area”, species-specific terms (“sheep or goat breeds AND Mediterranean area”, “sheep or goat cheese AND Mediterranean area”, “sheep or goat meat AND Mediterranean area”) and relevant terms (e.g., “Mediterranean landscape”, “climate change AND Mediterranean area”, “biodiversity”, “genetic resources”, “resilience”, “pasture management AND Mediterranean area”, “ecosystem services AND Mediterranean area”, smart technologies AND Mediterranean area, “Gentile di Puglia”).

**Figure 1:**
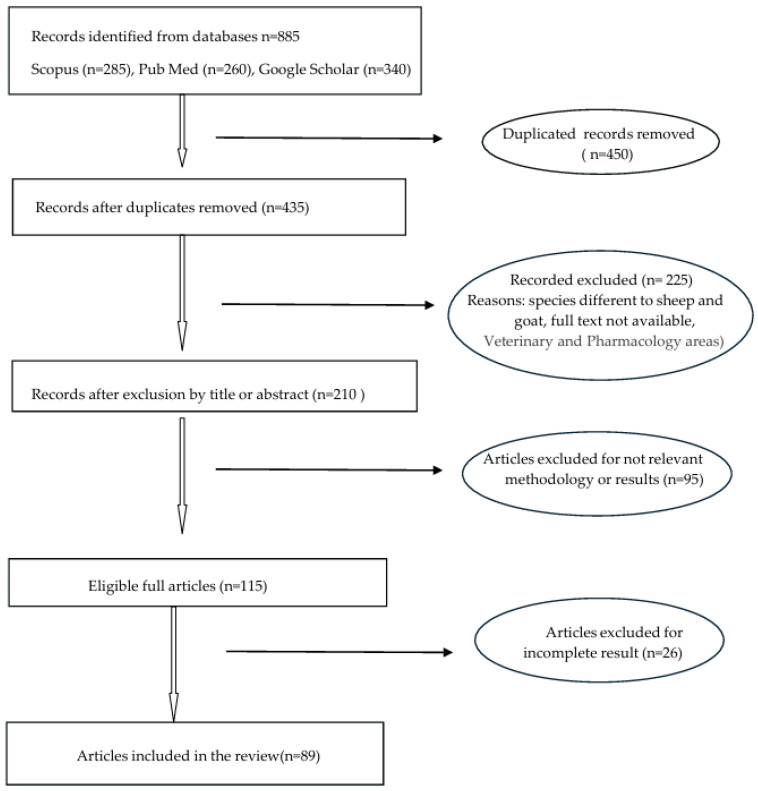
PRISMA-style flow chart of the literature search and study selection process.

A total of 885 articles were identified from the databases and 91 articles were considered to draft the review; the selection process is summarized in a PRISMA-style flow chart (Figure 1).

## 3. Livestock Farming in Marginal Areas of the Mediterranean Region

### 3.1. Sheep and Goats Population

The world’s small ruminant population is estimated at 2.551 billion heads, consisting of 1.363 billion sheep and 1.188 billion goats [16]. The distribution of sheep and goat populations in the Mediterranean area is shown in Table 1. With about 153 million heads of sheep and 40 million heads of goat, the Mediterranean accounts for around 15% of the world’s small ruminant population. It is interesting to note that in almost all Mediterranean regions, sheep numbers represent more than half of the ruminants, underscoring their relevance; this percentage is higher than the percentage reported by Dubeuf [17] referring to 2016. Indeed, even if some Mediterranean countries such as Italy, France, and Greece showed a decrease in sheep numbers [10,18], in others such as Algeria and Morocco, there was an increase, with the dominant sheep populations steadily increasing [19]. Sheep and goat populations are mainly distributed in Turkey and Algeria, where there are extensive production systems, while in the European Mediterranean countries, semi-extensive production systems are more widespread.

The diverse landscapes of the Mediterranean have fostered significant animal genetic diversity manifested by a large number of local breeds [20].

**Figure 2:**
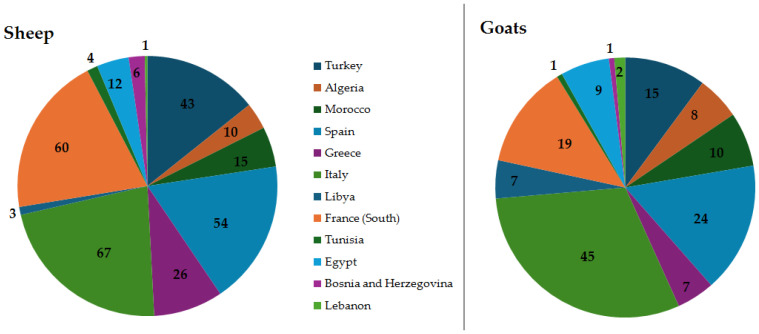
Number of local breeds within Mediterranean countries (FAOSTAT, 2025) [16].

Figure 2 shows the current number of breeds within Mediterranean countries (https://dadis-breed-datasheet-ws.firebaseapp.com; accessed on 26 February 2026); the list of the distinct breeds is reported in Supplementary Data (Table S1).

It is apparent that there is a great variety of sheep and goat breeds in the Mediterranean area; the higher number of breeds is evident in Italy and in France compared to other Mediterranean countries. In particular, Italy hosts remarkable ovine biodiversity, and this genetic richness is matched by environmental heterogeneity and by a variety of traditional management systems that reflect both environmental constraints and cultural heritage [21,22,23]. These breeds, often raised in extensive systems situated in marginal areas, have developed distinct adaptive traits that allow them to survive, reproduce, and remain productive under sub-optimal or highly variable conditions [24]. An updated and comprehensive characterization of the genetic diversity and structure of Italian goat populations was performed by Bionda et al. [25]. This study provides insights into adaptive genetic variation in goat species, and identifies populations and regions at greatest risk, emphasizing the need for targeted conservation and management strategies to preserve this unique component of livestock biodiversity.

### 3.2. Small-Ruminant Production

Mediterranean countries have created a longstanding and diversified tradition of fermented milk and fresh or ripened cheeses that are also the origin of a rich cooking heritage passed down through generations [26,27]. Mediterranean cheeses reflect the territory, climate, and traditions of each region; the pedoclimatic conditions also determine the botanical composition of the grass contributing to the cheeses’ flavour [28,29]. It is worth noting that although many traditional cheeses are still made in rural areas through artisanal methods, innovative processes for cheesemaking are applied in order to enhance the appeal of traditional cheeses in the market and among different types of consumers. An overview on specificities and novelties of the cheeses of the Eastern Mediterranean area was provided by Kalit et al. [30]. In particular, the authors highlighted the modern processes applied in cheesemaking in order to respond to changing consumer preferences, including advanced methods of geographical identification and authenticity testing. In recent years, the use of vegetable rennet in cheesemaking has increased to satisfy the growing requests from vegetarian consumers. Ritota et al. [31] evaluated, from a nutritional point of view, the use of aqueous extracts from typical thistles of the Mediterranean area in four different typical cheeses, Caciofiore (from Italy), Torta del Casar (from Spain), Queso de Murcia al vino (from Spain) and Feta (from Greece). These authors highlighted that all studied cheeses were good sources of essential nutrients such as calcium, vitamin A, phosphorus and zinc, with an optimal Ca/P molar ratio that makes calcium and phosphorus easily absorbable by the body. The combination of traditional cheesemaking methods with innovative approaches, such as the incorporation of probiotics in the sheep scamorza cheese, provides the possibility of developing novel products with beneficial nutritional features and peculiar sensory characteristics, particularly appreciated by consumers [32,33].

Among countries of the Mediterranean area, France, Greece, Italy, and Spain have a productive dairy sheep industry based on the use of local breeds and crossbreeds raised under semi-intensive systems for producing Protected Designation of Origin (PDO) cheeses according to traditional recipes [34]. These cheeses are currently appreciated as ingredients of healthy diets, since they have a high content of omega-3 polyunsaturated fatty acids (PUFA n-3) and conjugated linoleic acid (CLA) due to grazing systems that allow for milk with beneficial fatty acids [35,36,37].

PDO cheeses made from sheep milk such as Roquefort, Corsica, Pecorino, Manchego, Roncal, and Feta, recognized by the EU, play a major role in the international cheese trade and have a growing international market.

Dairy goat production in European Mediterranean countries is based on local breeds raised under more extensive systems than sheep. Goat milk is mainly processed on the farm into dairy products for national markets, but some PDO goat milk cheeses (e.g., Murcia al Vino) are exported. As suggested by Pulina et al. [34], to further develop the dairy sheep and goat sectors in the main Mediterranean dairy producing countries, some strategic priorities should be taken into account, such as: (i) genetic improvement and the use of new technologies at the farm level to increase ewes’ productivity; (ii) public support for farmers to adapt to milk price volatility; and (iii) the diversification of dairy products to increase the demand and sustain milk prices should be taken into account.

The distinctive traits and the nutritional features of sheep and goat dairy products from the Apulia region (Italy) are reported in Santillo et al. [38]. Currently, one PDO and four Traditional Agri-food Product (TAP) cheeses from milk of local sheep and goat breeds are recognized in the Apulia region: (i) canestrato pugliese, a PDO cheese manufactured from sheep milk; (ii) pecorino foggiano and scamorza di pecora, TAP cheeses manufactured from sheep milk; (iii) cacioricotta cheese, a TAP cheese made with sheep and goat milk; and (iv) caprino, TAP cheese made from goat milk. In particular, these TAPs can add value to the local dairy supply chain through both an enhanced quality of products and enhanced local supply chain resilience with short supply chains reducing logistics costs and increasing consumer trust. As reported by Celano et al. [39], caprino, pecorino, and cacioricotta cheeses have unique volatile organic compounds, allowing for a differentiated marketing approach compared to industrial dairy products that can contribute to valorising and safeguarding these TAP cheeses, sustaining local farming.

Related to meat production, sheep and goat meat consumption is deeply rooted in Mediterranean culture, characterized by high consumption of light lamb and kid, often during religious holidays like Easter or Christmas. Although, in recent years, the demand for sheep and goat meat in the Mediterranean area has increased, the trend is different according to the region. In particular, in Southern Europe, the consumption of small ruminant meat decreased, while, in Turkey and Lebanon the consumption is still higher than in Europe but lower than in Northern Africa where sheep and goat meat consumption is the highest [17]. A study involving many countries of the Mediterranean area (Italy, France, Spain, Greece, and Turkey) was conducted by Mandondolesi et al. [40] to explore the factors that affect both consumer and non-consumer perceptions of sheep and goat meat. The authors highlighted that consumers perceived sheep and goat meat as “tasty”, “natural” and “healthy” because of its lower environmental impact and fat content compared to other meats; in contrast, non-consumers considered this meat as not healthy and not satisfying.

Meat products constitute one of the ancient cultural heritages of Mediterranean countries. The most popular traditional meat products of the North African and Mediterranean countries were listed by Gagaoua et al. [41]; in this review, 32 ethnic meat products were presented, and except for one product, all of these products can be prepared using lamb meat.

An overview on Mediterranean cured meat products was published by Lorenzo et al. [42] to characterize traditional meat products within each geographic region. In particular, the authors highlighted that cured sheep and goat meat, in marginal Mediterranean areas, serve not just as delicacies but as traditional and practical solutions to food preservation and protein.

## 4. SWOT Analysis on Key Features of Mediterranean Livestock Systems

**Figure 3:**
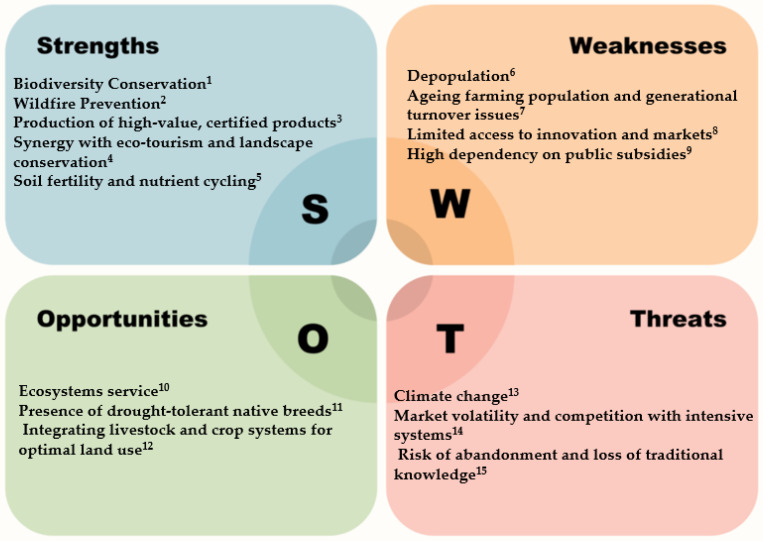
SWOT analysis related to key features of the Mediterranean livestock farming systems. ^1^ [9,43,44]; ^2^ [45,46,47,48,49]; ^3^ [50,51]; ^4^ [43,44]; ^5^ [52,53,54]; ^6^ [8,55]; ^7^ [56]; ^8^ [57,58]; ^9^ [59]; ^10^ [60,61] ^11^ [23] ^12^ [62] ^13^ [63,64] ^14^ [65] ^15^ [23,66].

The identification of the interactions between the key features of the livestock farming systems in the Mediterranean area and the possible strategies for its development represents a challenge and an opportunity for these rearing systems. Therefore, an ex ante evaluation, the Strength, Weaknesses, Opportunities, and Threats (SWOT) analysis, of livestock farming in the Mediterranean Region was performed. The SWOT analysis of livestock farming in the Mediterranean Region (Figure 3) summarizes the key points discussed above, categorizing them into strengths, weaknesses, opportunities, and threats related to the recent trends and challenges of Mediterranean livestock systems.

### 4.1. Strengths

Biodiversity conservation based on a high level of adaptation of local breeds to Mediterranean environments is one of the main strengths of the Mediterranean livestock systems. Local breeds are strongly linked to their territories, which contributes to landscape management, prevents land abandonment, and supports traditional economies based on high-quality niche products [43,44]. In particular, certified products such as PDO/PGI/TPA cheeses can create synergy with eco-tourism that represents an important resource for marginal areas of Mediterranean countries for promoting landscape conservation [50]. The importance of geographical certification products to farm income and rural economies was highlighted by Schimmenti et al. [51]. These authors examined the case of Pecorino Siciliano PDO and suggest that the production and the commercialization of the pecorino is a promising strategy to differentiate and increase the quality of dairy farm companies’ products and to improve the financial performance of producers, with foreseeable positive repercussions in the socioeconomically less favoured rural areas where they are located.

As reported by Tapaloga et al. [9], a multidisciplinary strategy that combines new biotechnologies with traditional knowledge and strong policy is necessary to preserve the long-term viability of livestock genetic resources.

Pastures represent one of the most significant ecological components of Mediterranean landscapes and provide important ecosystem functions [67]. Grazing by small ruminants is three times more effective than cattle in preventing shrub encroachment, maintaining open landscapes and aiding fire prevention [45,60]. Due to climate change, the frequency and intensity of wildfires has increased; as a consequence, promoting agro-pastoral activities is increasingly advocated in order to reduce fire hazard and improve landscape fire regulation capacity [46,47,48]. Heindorf et al. [49] highlighted how in the Mediterranean area, animal farming systems have the potential to mitigate wildfire disasters while synergistically providing co-benefits such as local products and enhanced biodiversity. These authors apply a biocultural lens to demonstrate how animal farming systems in the Mediterranean area can contribute to biodiversity conservation, climate resilience, and cultural values. In addition, rearing systems based on pasture contribute to the production of animal manure, adding organic matter to soils and improving fertility in these low-input systems, which is fundamental for soil fertility and nutrient cycling. The positive effect of extensive sheep grazing on soil fertility and vegetation control has been reported in several papers [52,53,54], in which it is highlighted that small-ruminant grazing impact soil physical properties and soil microorganisms.

### 4.2. Weakness

Many studies highlighted that in recent years, young people have been migrating from rural regions to urban centres, leaving behind ageing populations with limited capacity to maintain herding traditions; as a consequence, depopulation is widespread [8,55,57]. The risk of abandonment could be averted with the involvement of youth in targeted training and financial incentives that can attract new entrants, thus encouraging generational turnover [56].

Due to land abandonment and rural exodus, particularly in southern European countries, the traditional rural mosaic is disappearing, leading to high biomass content with increased frequency and intensity of wildfires [68]. An environmental management experiment to control wildfires in the mid-mountain Mediterranean area through shrub clearing to generate mosaic landscapes is reported by Lasanta et al. [69]. These authors, nearly four decades after the first interventions to convert abandoned land to grasslands, reported a significant reduction in the area affected by wildfires, a threefold increase in grazing area, and a fourfold increase in livestock numbers [70].

Generally, small-ruminant production systems are characterized by low competitiveness; small-scale producers often lack access to markets and innovation [57]. To ensure the sustainability of livestock farming in marginal Mediterranean areas, agricultural policies would better support the multifunctional role of pastoralism through market incentives and promoting value-added products. An overview of the economic performance of pastoral farms rearing sheep in seven Mediterranean countries (Algeria, Croatia, Cyprus, Greece, France, Italy, and Turkiye) was conducted by Skordos et al. [58] and revealed significant differences between countries and types concerning farm organization structure, use of local resources and choice of activities. Skordos et al. [58] highlighted that the role of income support was very important in EU pastoral systems, while for the non-EU countries, local markets played a more important role for product sales. In addition, rural communities would be actively involved in land-use planning and policy-making to ensure long-term sustainability in order to reduce the high dependency on public subsidies [59].

In recent years, digitalization has become an integral part of animal husbandry production. Utilization of smart technologies, such as precision livestock farming (PLF), in extensive livestock farming can provide real-time results on the conditions of grazing animals and their interaction with climate change [71,72]. However, some technologies have limitations, especially when applied to sheep grazing farming systems [73]. The virtual fencing technology allows livestock management and monitoring without physical boundaries, improving pasture productivity and biodiversity conservation and can mitigate the risk of predation on grazing livestock. The high cost, high learning variability between animals and lack of technological infrastructure in sheep and goat farms represent weaknesses for the application of this technology [74]. Sensor costs are higher than those for large animals due to miniaturization and lower production numbers, sheep and goat farms are often in mountainous and remote areas with poor technological infrastructure and internet services [75].

In addition, limited farm economic stability, trust in new technologies, and openness to innovation among farmers, usually of advanced age, can limit the adoption of PLF in Mediterranean small-ruminant farming [74].

### 4.3. Threats

Market volatility and competition with intensive system can be a threat for Mediterranean livestock farming. Tampaki et al. [65] highlighted that livestock farmers feel useful from having more information and training concerning market conditions in order to promote their products, suggesting that a promising strategy for the sustainability of livestock farms may be market differentiation and the public awareness of the nutritional value of products derived from local breeds.

Georgopoulou et al. [63] conducted a very detailed and integrated assessment of the effects of climate change on Greek agriculture and on socio-economic implications of these effects on output, highlighting that the direct economic losses in Greek crops and livestock are significant depending on the intensity of climate change.

It is worth noting that sheep and goats demonstrate a greater resilience to climate extremes, in terms of production, reproduction, and resistance to diseases, and are less competitive with humans for crops and grains than cattle [64]. As a result, the importance of sheep and goat farming increases gradually in comparison with cattle farming in both rural and industrialized countries. Therefore, the efforts of research and production must be focused on finding management practices such as feeding strategies for sustaining flock welfare and on raising the profitability of sheep and goat production during the summer season, turning, in this way, what could be a threat into an opportunity.

Pastoralism and transhumance are deeply embedded in the culture and traditions of Mediterranean countries and are part of their cultural heritage. Transhumance holds significance as cultural heritage, being indeed recognized as ‘Intangible Cultural Heritage of Humanity’ by UNESCO in 2023 for its role in maintaining landscape, knowledge and traditions [76]. Despite its cultural contribution, transhumance is currently carried out more and more rarely, contributing to the loss of traditional knowledge [66,77].

Ocak-Yetişgin and Canan [78] highlighted that pastoralists continue to engage in strategic adaptations, such as rotational grazing, breed diversification, and labour reorganization, grounded in longstanding ecological knowledge. This author demonstrated that transhumance can persist not as a relic of the past, but as a dynamic and ecologically efficient livelihood system capable of responding to multi-scalar rural challenges.

### 4.4. Opportunity

Ecosystem services provide a significant opportunity for Mediterranean livestock farming leading to transition from a solely production-focused model to a sustainable, multifunctional system that is recognized for its positive environmental and social impacts [60,61].

Drought-tolerant native breeds are prioritized for conservation and use in breeding resilient lines. A considerable cost to livestock production is represented by fighting and controlling endemic diseases and other stressors due to climate change, so there is a need for robust and easy-care animals that are able to thrive in particularly challenging environments. Serranito et al. [23] emphasize that local breeds are invaluable resources for environmental adaptation in the context of climate changes.

Integrated crop–livestock systems create synergies between crops and livestock that ensure the recycling of nutrients, minimize by-product waste, reduce external inputs and encourage sustainable resource management. Furthermore, this approach offers multifaceted benefits such as resource utilization and efficiency, increased production, climate resilience and preservation of biodiversity [62]. However, it is essential to have productive grasslands that ensure a continuous supply of high-quality forage to livestock systems; on the contrary, pasture degradation compromises the nutritional value of forage and reduces the carrying capacity of ecosystems with consequences on animal performance and on overall productivity of silvo-pastoral systems. In this context, the use of remote sensing technologies represents a powerful and cost-effective tool for quantifying vegetation dynamics, identifying degradation patterns, and supporting sustainable management decisions [67].

## 5. Gentile di Puglia Breed: An Example of Resilience

**Figure 4:**
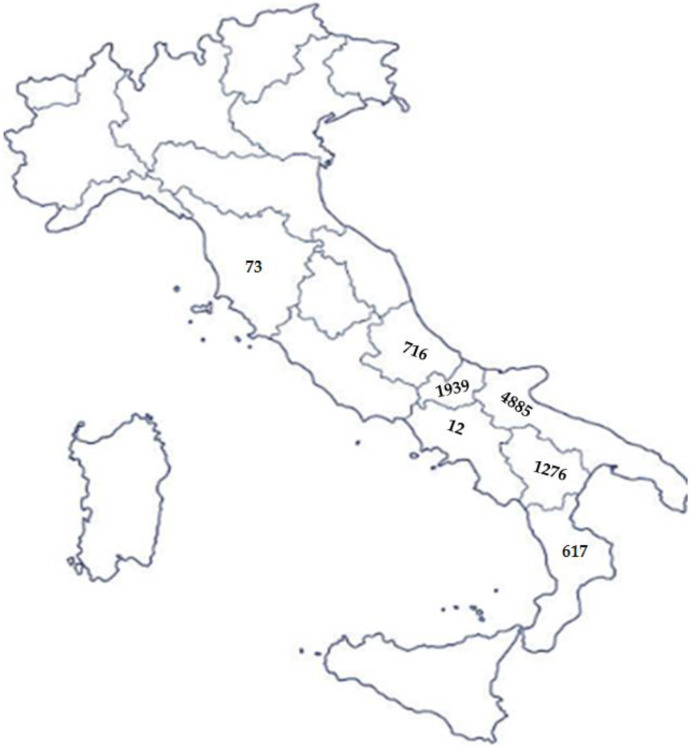
Distribution of Gentile di Puglia breed (number of heads) in Italy.

Gentile di Puglia is a millenary autochthonous sheep breed of the Southern Italy territory. It made up a total of 9505 heads on the national soil with 4855 heads in Apulia region, mainly located in the Tavoliere delle Puglia (an area around Foggia), and the remaining flocks were distributed in the neighbouring regions as reported in Figure 4 (https://www.vetinfo.it/j6_statistiche/#/report-pbi/89; accessed on 27 January 2026). It is worth noting that in recent years (from 2017 to date), the population of Gentile di Puglia has remained almost stable, ranging from 10,659 (in 2020) to 9153 (in 2024).

The Gentile di Puglia sheep, originally selected for fine wool production, are currently raised for multipurpose production providing ecosystem services, thus supporting not only agricultural productivity but also ecosystem resilience and rural socio-cultural activities. The main features of different types of services provided by the Gentile di Puglia breed are outlined in Table 2.

The milk contains high levels of protein (6–10%) and fat (8–11%) showing a high cheese yield, and it is destined for the production of traditional cheeses like Pecorino and Scamorza [32,79]. Albenzio et al. [33] highlighted that combining traditional methods with innovative processes such as the incorporation of probiotics in the Scamorza cheese made from Gentile di Puglia milk offers the possibility of developing novel products, potentially increasing the appeal of traditional products in the market and among different types of consumers. In addition, from the nutritional point of view, milk from Gentile di Puglia shows a better fatty acid profile compared to other Italian breeds, and has a lower content of saturated fatty acids than Altamurana and Sarda [80].

Meat is produced from lambs slaughtered at 30–45 days to meet the market demand. It is characterized by valuable nutritional features which are well documented by numerous studies on the fatty acid profile, showing a healthy profile with a high polyunsaturated fatty acids percentage [81,82,83].

These positive results in terms of potential benefits for human health and consumers’ acceptance of Gentile di Puglia lambs may represent an opportunity for valorisation and promotion of this breed. In 2023, the Gentile di Puglia breed gained the Slow Food Presidium designation for preserving a unique and cultural heritage (Slow Food Foundation for Biodiversity, www.fondazioneslowfood.com; consulted on 15 December 2025).

Gentile di Puglia sheep produce an excellent wool quality characterized by ultrafine and fine fibre classes (between 18 and 22 microns) and good fleece homogeneity [14,84]. Gentile di Puglia sheep exhibited a significantly higher secondary follicle density and secondary-to-primary follicle ratio compared to the Sarda breed, indicating a finer wool structure [85]. Its added value lies in the filament’s distinctive knurling (“crimp”), which ensures elasticity and softness in the finished garment. These characteristics of Gentile di Puglia wool allow for its use in the fashion industry. In particular, Montagna et al. [86] provided a critical and proactive vision for the valorisation of wool, illustrating how the regeneration of this fibre can be a central element in building a sustainable and ethical future textile. The authors refer to the association “Pecore Attive”, an initiative dedicated to the recovery and enhancement of wool from the Gentile di Puglia breed. In the last decade, the breeders of Gentile di Puglia sheep under the Slow Food Presidium or the association “Pecore Attive” have become promoters of the development of products and experiences related to the wool textile sector, furthering the use of sustainable and local materials. Their work is based on the use and application of traditional and manual processing (such as carding) and transformation techniques (such as spinning, weaving and felting) to create innovative products that combine tradition and contemporary design. This example highlights how innovation can be applied to an autochthonous breed, transforming local resources into valuable products that can compete in the international market.

The feasibility of a possible conservation strategy for the Gentile di Puglia based on the innovative use of its wool for the production of quality garments, was investigated by Sardaro et al. [87]. These authors using an integrated methodological approach to analysed the consumers’ preferences, the penetration market of this innovative product and the new wool value for farmers. The results highlighted the possibility of good demand for such products, which would reduce the difference in gross margin between Gentile di Puglia and the standardized intensively farmed Comisana from 57% to 3%.

Therefore, the Gentile di Puglia breed needs to be preserved for the productive, historical, and cultural value that it represents for the Southern Italy community and worldwide biodiversity.

In the context of breed conservation, where the maintenance of unique genetic ancestral traits is a central goal, a recent study found several non-introgressed regions associated with environmental adaptation in Gentile di Puglia [88] thus emphasizing the need for germplasm conservation actions.

It is known that the conservation of genetic animal resources can be performed in situ and ex situ, depending on whether animals are kept within their natural environments or production systems.

Temerario et al. [89] indicated that traditional reproductive management leads to progressive offspring reduction, while ex situ biotechnological conservation strategies, through immature oocyte vitrification and in vitro maturation, can support in situ conservation, leading to in vitro embryo production and transfer. Such strategies could allow farmers to plan the maintenance or expansion of the number of animals with a controlled and reducible environmental impact.

**Table 2:** Ecosystem services provided by the Gentile di Puglia breed.

Ecosystem Services		Insight	References
Provisioning services	Dairy and meat products with nutritional properties	Slow Food Presidium	[31,32,81,82,83]
	Wool	Pecore Attive	[88]
Supporting services	Conservation of autochthonous resources	Germoplasma cryobank	[89]
	Resilience to local parasites and disease		[88]
Regulatory services	Soil erosion control in grazing system Prevention of fire risk Prevention of shrub encroachment	Landscape Conservation	[38]
Cultural services	Preservation of transhumance Tradition Promotion of agrotourism activity	Cultural identity of South Italy linked to pastoralism	[12]

Alongside the economic valorisation of production, there is also a profound socio-environmental value. The Gentile di Puglia breed is raised in marginal areas (Monti Dauni, Gargano, Murgia), areas at risk of depopulation. The breeder is not only a shepherd, but also a “guardian of the land” who prevents hydrogeological instability and all of the problems associated with the abandonment of rural areas. Purchasing the finished product, therefore, also supports the protection of these contexts. Higher prices on Gentile di Puglia products can be achieved by featuring their positive environmental impact and promoting their quality properties.

## 6. Conclusions

Small-ruminant farming systems of Mediterranean regions are crucial in conserving biodiversity, providing dairy products with appreciated nutritional and sensorial properties, creating multifunctional landscapes and preserving traditional knowledge and cultural heritage. The potential of small-ruminant farming is not completely unlocked within landscapes in Mediterranean regions. Promoting knowledge and public awareness of the vital role of extensive pastoral practices for landscape conservation level could create new marketing opportunities for local small-ruminant products. Higher prices for these products can be achieved by featuring their positive environmental impact and promoting their quality aspects. Additionally, the poor professional profile of livestock workers could be upgraded, possibly increasing the attractiveness of their professions, especially in the younger generations. The focus on the Gentile di Puglia breed highlights that innovation can be applied to autochthonous sheep livestock systems, transforming local resource into valuable products.

## Figures and Tables

**Table 1:** Sheep and goat populations in the Mediterranean area (FAOSTAT, 2025) [16].

	Small Ruminant Numbers (Million Heads)		Small Ruminants as % of Total of Ruminants *	
Country	Sheep	Goats	Sheep	Goats
Turkey	44.7	12.3	79.7	19.4
Algeria	32.4	5.08	82.5	12.9
Morocco	19.6	6.3	69.3	22.4
Spain	15.6	3	59.9	12.7
Greece	8.5	4.4	71,0	23.5
Italy	5.6	1	43.1	7.3
Libya	7.5	2.8	71.9	26.1
France (South)	7	1.2	27.1	5.5
Tunisia	6.9	1.7	27.0	6.7
Egypt	1.95	1.2	27.3	15.9
Bosnia and Herzegovina	1.1	0.1	71.2	4.0
Lebanon	0.6	0.4	50.3	40.8

* Cattle, buffalo, sheep and goat.

## Data Availability

The original contributions presented in this study are included in the article/Supplementary Materials. Further inquiries can be directed to the corresponding author.

## Supplementary Materials

The following supporting information can be downloaded at https://www.mdpi.com/article/10.3390/ani16091356/s1: Table S1: List of sheep breed reared in the main Mediterranean countries; Table S2: List of goats breed reared in the main Mediterranean countries.
