# Supplementary material for: An Analysis of Small-Ruminant Farming in Marginal Area of the Mediterranean Region: A Focus on the Gentile di Puglia Breed
Source: Animals (Basel). 2026 Apr 28;16(9):1356. doi: 10.3390/ani16091356 (PMC13163137; doi:10.3390/ani16091356)
Supplement: Supplementary file 1 [file animals-16-01356-s001.zip › animals-4230498-supplementary.pdf]

SHEEP											
**ITALY**	**SPAIN**	**Greece**	**France**	**Morocco**	**Turkey**	**Egitto**	**Algeria**	**Lybia**	**Tunisia**	**Bosnia and Herzegovina**	**Lebanon**
Alpagota	Alcarreña	Agriniou	Aure et Campan	Beni Ahsen	Acıpayam	Aboudleik	Barbarine	AWASSI	Barbarine	Dubska	Awassi
Altamurana	Ansotana	Anogeiano	Avranchin	Beni Guil	Akkaraman	Barki	Berbère	Libyan	Noire de	Kupreska	
Appenninica	Aranesa	Argos	Barégeoise	Boujaad	Anadolu Merinosu	Ebeidi	D'man	TEHRI	Queue Fine	Podveleska	
Assaf	Assaf	Boutsiko	Basco-Béarnaise	Caussu du Lot	Anatolian Red	Fallahi	Hamra		Sicilo-Sarde	Privorska	
Bagnolese	Berrichon du Cher	Chios	Belle Ile	D'man	Asaf	Farafra	Ouled Djellal			Sjenicka	
Barbaresca Sicilia	Canaria	Florina	Berrichon de	DS	Bandırma	Kanzi	Rembi			Stolacka	
Bergamasca	Canaria de Pelo	Friesian	Berrichon du	Ile de France	Cine Capari	Maenit	Sidahou				
Berrichonne du Cl	Carranzana	Frizarta	Bizet	Lacaune	Dağlıç	Ossimi	Taadmit				
Biellese	Carranzana (variedad Negra)	Kalarritiko	Blackface	Ouled Jellal	Gökçeada	Rahmani	Tazegzawt				
Brentegana	Cartera	Karagouniko	Blanc du Massif	Sardi	Güney Karaman	Saidi					
Brianzola	Castellana	Karystou	Bleu du Maine	Timahdite	Halkali	Sanabawi					
Brigasca	Castellana (variedad Negra)	Katafygiou	Boulonnaise	Blanche de	Hamdani	Sohagi					
Brogne	Chamarita	Katsika	Brigasque	Merinos	Hasak						
Ciavenasca	Charmoise	Kefallinias	Castillonnaise	Noire de	Hasmer						
Ciuta	Churra	Kymi	Caussenarde des	Saghro	Hemşin						
Comisana	Churra Lebrijana	Lesvos	Causses du Lot		Herik						
Cornella Bianca	Churra Tensina	Pilioritiko	Charmoise		Ivesi						
Cornigliese	Colmenareña	Roumloukiou	Clun Forest		Kamakuyruk						
Dell'Amiata	Fleischschaf	Sarakatsaniko	Corse		Kangal Akkaraman						
Delle Langhe	Guirra	Serrai	Cotentin		Karacabey Merino						
Fabrianese	Lacaune	Sfakiano	Dorset Down		Karagül						
Finarda	Landschaff	Sitia	Est à laine		Karakas						
Frabosana	Latxa	Skopelos	Finnoise		Karakaçan						
Frisona	Lojeña	Thraki	Grivette		Karayaka						
Garessina	Maellana	Vlahiko	Hampshire		Karya						
Garfagnina Bianc	Manchega	Zakynthos	INRA 401		Kıvırcık						
Gentile Di Puglia	Manchega (variedad Negra)		Ile-de-France		Malya						
Ile de France	Merina		Lacaune Lait		Menemen						
Istriana	Merina (variedad Negra)		Lacaune Viande		Morkaraman						
Juraschaf	Merina (variedad de los Montes Universales)		Landaise		Norduz						
Lacaune	Merina de Grazalema		Landes de		Of Koyunu						
Lamon	Merino Precoz		Limousine		Orta Anadolu						
Leccese	Montesina		Lourdaise		Pırlak						
Marrane	Navarra		Manech Tête		Ramlıç						
Massese	Ojalada		Manech Tête		Sakız						
Matesina	Ojinegra de Teruel		Martinik		Sönmez						
Merinizzata Italia	Ovella Eivissenca		Montagne noire		Tahirova						
Nera di Arbus	Ovella Galega		Mourerous		Tuj						
Nostrana	Ovella Mallorquina		Mouton		Türkgeldi						
Noticiana	Ovella Menorquina		Mouton vendéen		Zom						
Pecora di Corteno	Ovella Roja Mallorquina		Mérinos d'Arles		Çepni Koyunu						
Pinzirita	Palmera		Mérinos de		Çine Çaparı						
Plezzana	Rasa Aragonesa		Mérinos précoce		Ödemis						
Pomarancina	Ripollesa		Noir du Velay								

Pusterese	Roya Bilbilitana		Ouessant								
Quadrella	Rubia del Molar		PréAlpes du Sud								
Romanov	Salz		Raiole								
Rosset	Sasi Ardi		Rava								
Saltasassi	Segureña		Romanov								
Sambucana	Talaverana		Rouge de l'ouest								
Sampeirina	Tudelana		Rouge du								
Sarda	Xalda		Roussin de La								
Savoiarda	Xisqueta		Sasi Ardi								
Schwarzbraunes	Île de France		Shropshire								
Schwarznasenschaf			Solognote								
Sciara			Southdown								
Sopravissana			Suffolk								
Tacola			Tarasconnaise								
Texel			Texel								
Tiroler Steinschaf			Thônes et								
Trimeticcia di Segezia											
Turchessa											
Valle del Belice											
Varesina											
Vicentina											
Villonesser Schaf											
Zerasca											

Table S1-List of sheep breed reared in the main Mediterranean countries

GOAT											
**ITALY**	**SPAIN**	**Greece**	**France**	**Morocco**	**Turkey**	**Egitto**	**Algeria**	**Lybia**	**Tunisia**	**Bosnia and Herzegovina**	**Lebanon**
Alpina	Alpina	Alpine	Alpine	Alpine	Abaza	AHS	Arabe	CYPRUS	Arbi	Bosnian	Damasc
Argentata dell'Etna	Azpi Gorri	Damascus	Angora	Murciana	Ankara	Baladi	Arabia	KURDI			Mambe
Bianca Monticellana	Bermeya	Local Breeds	Blanche des	Noire	Bezoar	Barki	Berber	Libyan			
Bionda dell'Adamello	Blanca Andaluza o Serrana	Malta	Boer	Saanen	Gürcü	Black	Kabyle	MOURETHA			
Camosciata delle Alpi	Blanca Celtibérica	Saanen	Catalan	Barcha	Halep	Saidi	Makatia	SHAMI			
Campobasso Grigia Molisana	Blanca de Rasquera	Skopelos	Chèvre de	beni	Honaml	Wahati	Mzabite	TARGI			
Capestrina	Cabra Galega	Toggenburg	Chèvre des Fossés	Aarous	Ispir	XXXXX	Sahelia	TEBAWI			
Capra Istriana	Cabra de las Mesetas		Chèvre des Savoie	Draa	Kaçkar	XXXXX	Tuareg				
Capra dell'Aspromonte	Del Guadarrama		Chèvre du Massif	El Hamra	Kilis	Zaraibi					
Cilentana Fulva	Eivissenca		Corse	Ghazalia	Kıl Keçi						
Cilentana Grigia	Florida		Cou-Clair de		Mahalli						
Cilentana Nera	Jurdana		Créole		Malta						
Ciociara Grigia	Majorera		Peï		Mingrel						
Di Benevento	Malagueña		Poitevine		Norduz						
Di L'Aquila	Mallorquina		Provencale		Türk						
Di Montecristo	Moncaína		Pyrénéenne								
Di Potenza	Murciana-Granadina		Rove								
Di Teramo	Negra Serrana		Saanen								
Fiurinà	Palmera		Sundgau								
Frisa valtellinese	Payoya										
Garfagnina Bianca	Pirenaica										
Garganica	Retinta										
Girgentana	Tinerfeña										
Jonica	Verata										
Lariana o Di Livo											
Maltese											
Messinese											
Murciana											
Napoletana											
Nicastrese											
Orobica o Valgerola											
Passeirer Gebirgziege											
Pomellata											
Roccaverano											
Rossa Mediterranea											
Rustica di Calabria											
Saanen											
Sarda											
Sarda Primitiva											
Screziata											
Sempione											
Valdostana											
Vallesana											

Sempione											
Valdostana											
Vallesana											
Verzaschese											

Table S2-List of goats breed reared in the main Mediterranean countries
